# Endobronchial brachytherapy as definitive treatment for endobronchial metastasis after surgery of non-small cell lung cancer

**DOI:** 10.1186/s12957-021-02434-9

**Published:** 2021-11-07

**Authors:** Atsushi Ito, Daisuke Yamaguchi, Shinji Kaneda, Koji Kawaguchi, Akira Shimamoto, Makiko Kubooka, Yoshihito Nomoto, Motoshi Takao

**Affiliations:** 1 Department of Thoracic and Cardiovascular Surgery, Graduate School of Medicine, Mie University, 2-174 Edobashi, Tsu, Mie 514-8507 Japan; 2 Department of Radiology, Graduate School of Medicine, Mie University, Tsu, Japan; 3 Department of Radiation Oncology, Graduate School of Medicine, Mie University, Tsu, Japan

**Keywords:** Endobronchial metastasis, Endobronchial brachytherapy, Definitive treatment

## Abstract

**Background:**

Endobronchial metastasis is a very rare type of recurrence after lung cancer surgery. Surgical intervention may be difficult to perform due to the postoperative reduction in the activities of daily living (ADL) and the invasiveness associated with redo surgery. In such cases, endobronchial brachytherapy (EBBT) plays an important role not only as a palliative treatment, but also as a definitive treatment with curative intent.

**Case presentation:**

Three men (64, 69, and 74 years old) underwent combination therapy of external beam radiation therapy (EBRT) and EBBT for endobronchial metastasis after lobectomy of stage I–II non-small cell lung cancer (NSCLC): 2 cases of squamous cell carcinoma and 1 of adenocarcinoma. We used a special source-centralizing applicator for EBBT to avoid eccentric distribution of the radiation dose. Follow-up was considered to start from the end of brachytherapy. None of our patients experienced severe adverse events, and none needed extensive outpatient treatment. Local control was achieved in all cases by a bronchoscopic evaluation. All patients were alive after 31, 38, and 92 months of follow-up, respectively. In the adenocarcinoma patient, two metastases to the lung were discovered 3 years after EBBT, and the patient underwent partial wedge resection.

**Conclusions:**

EBBT may be a promising treatment with curative intent for endobronchial metastasis after surgery of NSCLC.

## Background

Despite the advances in radiation therapy, chemotherapy, and newly developed molecular-targeted therapies, lung cancer remains a major health problem and a leading cause of cancer mortality worldwide. The majority of mortalities in the case of postoperative non-small cell lung cancer (NSCLC) are due to the development of recurrence [1]. Endobronchial metastasis is a very rare type of recurrence defined in detail by Schoenbaum and Viamonte [2]. Patients with endobronchial metastasis often suffer from symptoms of airway obstruction, including coughing, hemoptysis, dyspnea, and wheezing. Therefore, removal of the bronchial obstruction can help to improve the patient’s clinical status. However, surgical intervention may be difficult due to the postoperative reduction in activities of daily living (ADL) and the invasiveness associated with redo surgery. In such cases, endobronchial brachytherapy (EBBT) plays an important role not only as a palliative treatment, but also as a definitive treatment with curative intent.

EBBT is the radiation therapy in which the radioactive material is targeted at the bronchial lumen carrying the target lesion. Historically, EBBT has often been used for palliative treatment and has been shown to be effective in improving bronchial stenosis and obstruction caused by tumors [3]. Recently, several studies have demonstrated that EBBT plays a limited but specific role in definitive treatment with curative intent in select cases of early endobronchial disease, in select advanced inoperable tumors combined with external beam radiotherapy (EBRT), or in the postoperative treatment of small, residual peribronchial disease [3–6].

We herein report our experience with EBBT as a definitive treatment for endobronchial metastasis after surgery of NSCLC to assess the feasibility and efficacy of this approach.

## Case presentation

Between 2013 and 2018, 3 men (64, 69, and 74 years old) underwent a combination therapy of EBRT and EBBT for endobronchial metastasis after lobectomy of stage I–II NSCLC: 2 cases of squamous cell carcinoma and 1 of adenocarcinoma. At the timing of the initial surgery, R0 resection was achieved in all cases. The diagnosis of cancer recurrence was made by a bronchoscopic biopsy. In all patients, surgical intervention was considered difficult due to postoperative reductions in the ADL and the invasiveness associated with redo surgery.

**Fig. 1:**
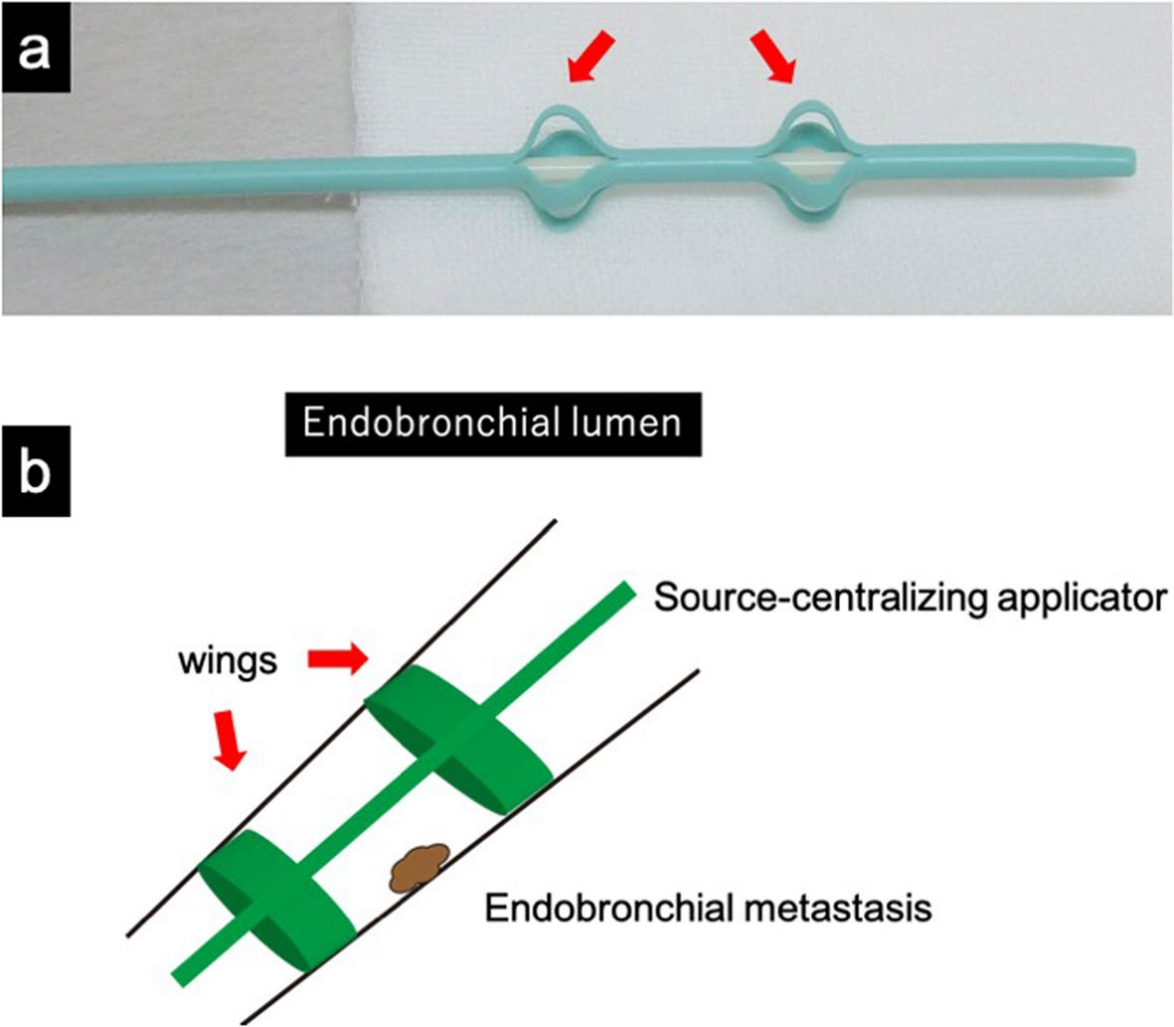
**a** A special source-centralizing applicator developed by Nomoto et al. Two self-expandable wings (arrow). **b** Schema of endobronchial brachytherapy using the special source-centralizing applicator. The applicator is adjusted to the optimal position such that the tumor is located between the two wings

The radiation doses of EBRT and EBBT were 40 Gy in 20 fractions and 18 Gy in 3 fractions. Brachytherapy was performed once per week using a high dose rate Ir-192 after-loading machine (microSelectron®, Nucletron, Elekta Co., Sweden). The reference dose points were 5–7 mm from the source axis, depending on the bronchial diameter. Chemotherapy was not performed, and EBRT was performed prior to EBBT. The aims of EBRT prior to EBBT are to reduce the tumor size and to obtain a uniform dose distribution to the bronchial wall. In order to keep the interval between radiation therapies as short as possible, EBBT was performed from the day after EBRT was completed. We used a special source-centralizing applicator (Create Medic Co. Ltd., Yokohama, Japan) developed by Nomoto et al. for EBBT to avoid eccentric distribution of the radiation dose (Fig. 1a) [7]. This applicator can hold the source-delivering catheter because the two self-expandable wings open according to the bronchial diameter [5]. The applicator is adjusted to the optimal position such that the tumor is located between the two wings (Fig. 1b) [5]. The distance from the source axis to the bronchial wall was measured on the planning computed tomography (CT) image [5].

Follow-up was considered to start from the end of EBBT. The local response was evaluated by bronchoscopy performed 1 to 2 months after the end of EBBT. In addition, CT was performed every 6 months for the first 2 years and then yearly for at least 5 years of follow-up.

### Case 1

**Fig. 2:**
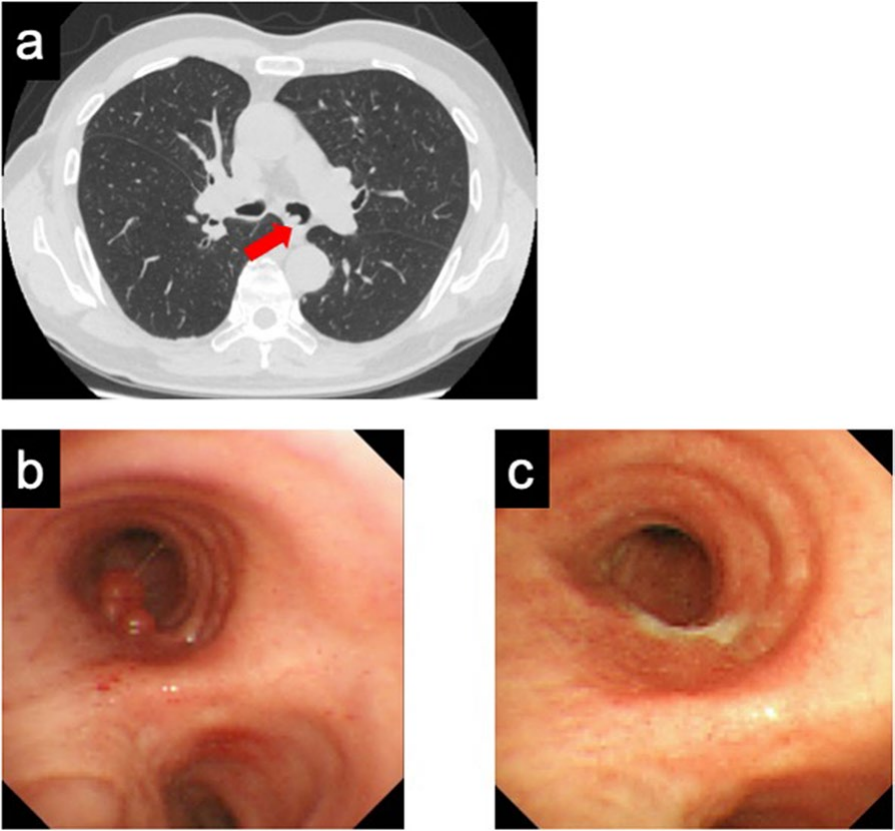
**a** CT findings showed the endobronchial metastasis at the left main bronchus (arrow). Bronchoscopic image of **b** before irradiation and **c** after irradiation

A 64-year-old man underwent the right upper lobectomy for lung cancer of adenocarcinoma (2.0 cm, pT1bN0M0, pStage IA2). The patient had no postoperative complications and was discharged on postoperative day 9. Nine years after the initial surgery, postoperative follow-up CT showed a left endobronchial nodule near the peripheral tracheal bifurcation (Fig. 2a). Bronchoscopy confirmed a protruding lesion emanating from the posterior wall of the left main bronchus (Fig. 2b). After a diagnosis of adenocarcinoma by tumor resection using a bronchoscopic snare was made, we performed a combination of EBRT and EBBT for the small residual disease as a definitive treatment. The patient had achieved a complete local response without adverse events at the time of the bronchoscopic evaluation (Fig. 2c). Three years after irradiation, CT revealed a new partly solid nodule at the left lower lobe. The patient then underwent partial wedge resection and is still alive at 90 months since the irradiation.

### Case 2

**Fig. 3:**
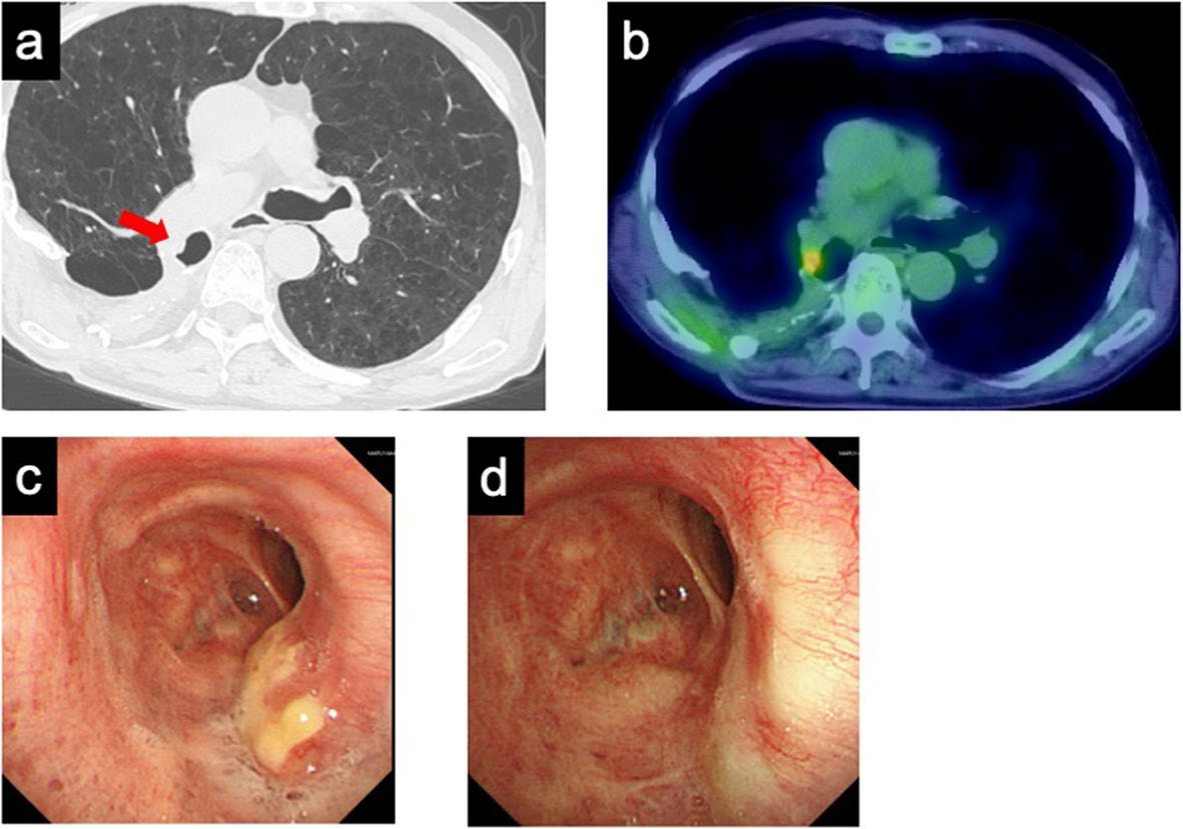
**a** CT findings showed the endobronchial metastasis at the truncus intermedius (arrow). **b** PET-CT revealed an uptake in the lesion [SUV_max_ 3.8]. Bronchoscopic image of **c** before irradiation and **d** after irradiation

A 69-year-old man underwent the right lower lobectomy for lung cancer of squamous cell carcinoma (4.0 cm, pT2aN0M0, pStage IB). Two weeks after the surgery, we performed a right-sided open-window thoracostomy due to a postoperative bronchopleural fistula. Three months after open-window thoracostomy, he was discharged after undergoing thoracoplasty with omental plombage. One year and 6 months after discharge, postoperative follow-up CT showed a right endobronchial nodule at the truncus intermedius (Fig. 3a). Fluorodeoxyglucose-positron emission tomography (FDG-PET) revealed an uptake at the lesion (maximum standardized uptake value [SUV_max_] 3.8) (Fig. 3b). After a diagnosis of squamous cell carcinoma was made by a bronchoscopic biopsy (Fig. 3c), we performed a combination of EBRT and EBBT for the endobronchial metastasis as a definitive treatment. The patient had achieved a complete local response without adverse events at the time of the bronchoscopic evaluation (Fig. 3d). The patient has remained disease-free for 36 months since the irradiation.

### Case 3

**Fig. 4:**
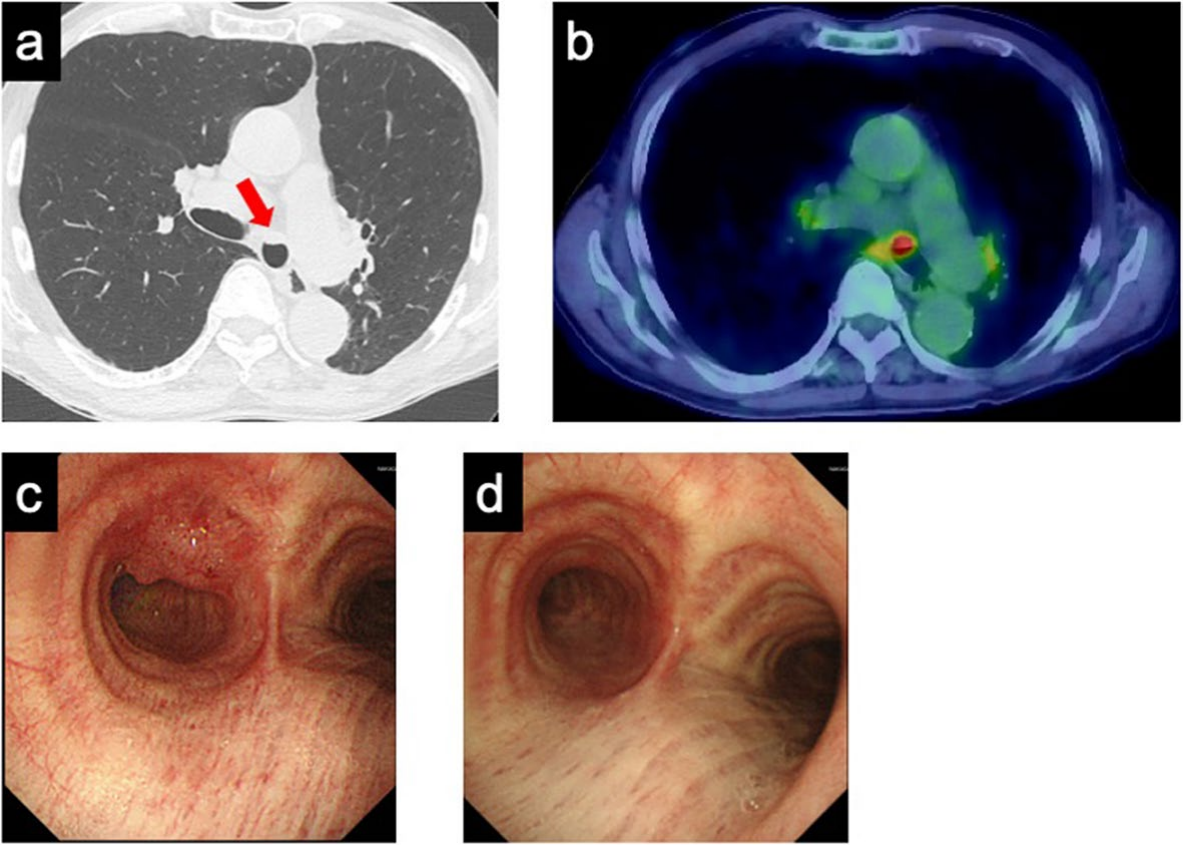
**a** CT findings showed the endobronchial metastasis at the left main bronchus (arrow). **b** PET-CT revealed an uptake in the lesion [SUV_max_ 7.4]. Bronchoscopic image of **c** before irradiation and **d** after irradiation

A 74-year-old man underwent the left lower lobectomy for lung cancer of squamous cell carcinoma (5.7 cm, pT2bN0M0, pStage IIA). The patient had no postoperative complications and was discharged at postoperative day 6. Two years after discharge, postoperative follow-up CT showed a left endobronchial nodule near the peripheral tracheal bifurcation (Fig. 4a). FDG-PET revealed an uptake at the lesion (SUV_max_ 7.4) (Fig. 4b). After a diagnosis of squamous cell carcinoma was made by a bronchoscopic biopsy (Fig. 4c), we performed a combination of EBRT and EBBT for the endobronchial metastasis as a definitive treatment. The patient had achieved a complete local response without adverse events at the time of the bronchoscopic evaluation (Fig. 4d). The patient has remained disease-free for 29 months since the irradiation.

## Discussion

The frequency of endobronchial metastasis from non-pulmonary malignancies is known to range from about 2 to 50%, varying by the definition of metastasis used and the types of cancer included [8–10]. The overall incidence of tracheal metastasis is 0.44% in surgically resected NSCLC, and the incidence of tracheal metastasis is lower in primary lung cancers than in non-pulmonary malignancies [10]. Hemoptysis with coughing is the most common symptom of endobronchial metastasis, with an incidence of 41–62% reported [10]. In our cases, all patients with endobronchial metastasis were free from symptoms despite the endobronchial nodule shown on postoperative follow-up CT. To determine whether or not these lesions are sputum, it is useful to examine the lesion uptake of FDG by PET-CT. In our cases, the uptake of FDG was revealed in endobronchial lesions, but there were no metastatic lesions noted outside the thorax, including in the brain.

Schoenbaum and Viamonte report that metastatic spread of malignant tumors to bronchi occurs by direct extension from mediastinal lesion, invasion of bronchus by parenchymal mass, or direct metastasis to the wall of the bronchi [2]. In our cases, endobronchial metastasis occurred despite the absence of lymph node metastasis, and the metastatic pathway was suspected of being the bronchial veins in the bronchial wall.

Surgery was not indicated in the present cases because extensive adhesions were expected on the postoperative side, and recurrence near the tracheal bifurcation might require extremely invasive and difficult surgery, such as carinal reconstruction or pneumonectomy. Therefore, we performed a combination of EBRT and EBBT as a definitive treatment, and none of our patients experienced local recurrence. The most serious adverse events of EBBT are fatal hemoptysis and severe bronchitis due to over-irradiation. These adverse events are caused by the radioactive source being placed at eccentric locations in the bronchial lumen, thereby leading to localized hot spots on the bronchial mucous membrane [3]. We therefore used a special source-centralizing applicator to avoid eccentric distribution of the radiation dose. One month after the end of EBBT, bronchoscopy revealed inflammation of the bronchial mucosa; however, none of our patients experienced such severe adverse events, and none needed extensive outpatient treatment. This applicator was also used to protect the bronchial mucosa from high-dose irradiation in the reports of Kawamura et al. [11], Hosni et al. [6], and Nomoto et al. [5].

There have been several reports regarding the combination of EBRT and EBBT therapy in either a curative or palliative setting. Nomoto et al. reported that the 3-year overall and progression-free survival rates were 79% and 77%, respectively, in 15 patients [5]. In addition, Hosni et al. reported that the 2-year overall survival and local control rates were 67% and 89%, respectively, in 23 patients [6]. While these reports describe relatively few patients, these outcomes suggest that EBBT may be expected to achieve excellent tumor control.

## Conclusions

EBBT is possibly a promising treatment with curative intent for endobronchial metastasis after surgery of NSCLC. In particular, the use of the special source-centralizing applicator can prevent over-irradiation of the bronchial mucosa and lead to safe EBBT without severe adverse events.

## Data Availability

Not applicable.

## Abbreviations

NSCLC: Non-small cell lung cancer
ADL: Activities of daily living
EBBT: Endobronchial brachytherapy
EBRT: External beam radiotherapy
CT: Computed tomography
FDG-PET: Fluorodeoxyglucose-positron emission tomography
SUV_max_: Maximum standardized uptake value
